# Crohn’s and Colitis Canada’s 2021 Impact of COVID-19 & Inflammatory Bowel Disease in Canada: A Knowledge Translation Strategy

**DOI:** 10.1093/jcag/gwab028

**Published:** 2021-11-05

**Authors:** Gilaad G Kaplan, Joseph W Windsor, Janet Crain, Lisa Barrett, Charles N Bernstein, Alain Bitton, Usha Chauhan, Stephanie Coward, Sharyle Fowler, Jean-Eric Ghia, Deanna L Gibson, Anne M Griffiths, Jennifer L Jones, Reena Khanna, M Ellen Kuenzig, Peter L Lakatos, Kate Lee, David R Mack, John K Marshall, Mina Mawani, Sanjay K Murthy, Remo Panaccione, Cynthia H Seow, Laura E Targownik, Sandra Zelinsky, Eric I Benchimol

**Affiliations:** 1 Department of Medicine, University of Calgary, Calgary, Alberta, Canada; 2 Department of Community Health Sciences, University of Calgary, Calgary, Alberta, Canada; 3 KTE Bridge Consulting, Toronto, Ontario, Canada; 4 Department of Medicine, Dalhousie University, Halifax, Nova Scotia, Canada; 5 Department of Internal Medicine, Max Rady College of Medicine, Rady Faculty of Health Sciences, University of Manitoba, Winnipeg, Manitoba, Canada; 6 University of Manitoba IBD Clinical and Research Centre, Winnipeg, Manitoba, Canada; 7 Department of Medicine, McGill University Health Centre, McGill University, Montreal, Quebec, Canada; 8 Hamilton Health Science, Department of Medicine and Farncombe Family Digestive Health Research Institute, McMaster University, Hamilton, Ontario, Canada; 9 Department of Medicine, University of Saskatchewan, Saskatoon, Saskatchewan, Canada; 10 Department of Immunology and Internal Medicine section of Gastroenterology, Max Rady College of Medicine, Rady Faculty of Health Sciences, University of Manitoba and University of Manitoba Inflammatory Bowel Disease Clinical and Research Centre, Winnipeg, Manitoba, Canada; 11 Department of Biology, Faculty of Science; Department of Medicine, Faculty of Medicine, The University of British Columbia, Okanagan campus, Kelowna, British Columbia, Canada; 12 SickKids Inflammatory Bowel Disease Centre, Division of Gastroenterology, Hepatology and Nutrition, The Hospital for Sick Children, Toronto, Ontario, Canada; 13 Child Health Evaluative Sciences, SickKids Research Institute, Toronto, Ontario, Canada; 14 ICES, Toronto, Ontario, Canada; 15 Department of Paediatrics and Institute of Health Policy, Management and Evaluation, University of Toronto, Toronto, Ontario, Canada; 16 London Health Sciences Centre-University Campus, Western University, London, Ontario, Canada; 17 1^st^ Department of Medicine, Semmelweis University, Budapest, Hungary; 18 Crohn’s and Colitis Canada, Toronto, Ontario, Canada; 19 CHEO Inflammatory Bowel Disease Centre and Department of Pediatrics, University of Ottawa, Ontario, Canada; 20 The Ottawa Hospital IBD Centre, Department of Medicine, University of Ottawa, Ottawa, Ontario, Canada; 21 Division of Gastroenterology and Hepatology, Department of Medicine, University of Calgary, Calgary, Alberta, Canada; 22 Division of Gastroenterology and Hepatology, Mount Sinai Hospital, University of Toronto, Toronto, Ontario, Canada

**Keywords:** Crohn’s disease, Ulcerative colitis, SARS-CoV-2, Coronavirus

## Abstract

The prevalence of inflammatory bowel diseases (IBD), Crohn’s disease and ulcerative colitis, in Canada, is over 0.75% in 2021. Many individuals with IBD are immunocompromised. Consequently, the World Health Organization’s declaration of a global pandemic uniquely impacted those with IBD. Crohn’s and Colitis Canada (CCC) formed the COVID-19 and IBD Taskforce to provide evidence-based guidance during the pandemic to individuals with IBD and their families. The Taskforce met regularly through the course of the pandemic, synthesizing available information on the impact of COVID-19 on IBD. At first, the information was extrapolated from expert consensus guidelines, but eventually, recommendations were adapted for an international registry of worldwide cases of COVID-19 in people with IBD. The task force launched a knowledge translation initiative consisting of a webinar series and online resources to communicate information directly to the IBD community. Taskforce recommendations were posted to CCC’s website and included guidance such as risk stratification, management of immunosuppressant medications, physical distancing, and mental health. A weekly webinar series communicated critical information directly to the IBD community. During the pandemic, traffic to CCC’s website increased with 484,755 unique views of the COVID-19 webpages and 126,187 views of the 23 webinars, including their video clips. CCC’s COVID-19 and IBD Taskforce provided critical guidance to the IBD community as the pandemic emerged, the nation underwent a lockdown, the economy reopened, and the second wave ensued. By integrating public health guidance through the unique prism of a vulnerable population, CCC’s knowledge translation platform informed and protected the IBD community.

**Key Points**

- During the COVID-19 pandemic, one of the most essential health services has been the communication of expert health information and population-level advice; for the IBD population, this was achieved through expert-created online materials and frequent webinars geared towards a public audience.
- Because the epidemiology of the COVID-19 pandemic differed by region, emphasis is placed on providing the best information possible so that people with IBD can assess their personal risk based on personal health risk factors, ability to stay home, and the state of local outbreaks.
- In addition to increased web content and topical webinars, one of the most effective tools at communicating expert information to the IBD population were short topical videos spliced from the full webinar series that allowed individuals to search and find answers to specific questions related to their personal risk and/or the COVID-19 pandemic.

## Introduction

**Figure 1.**
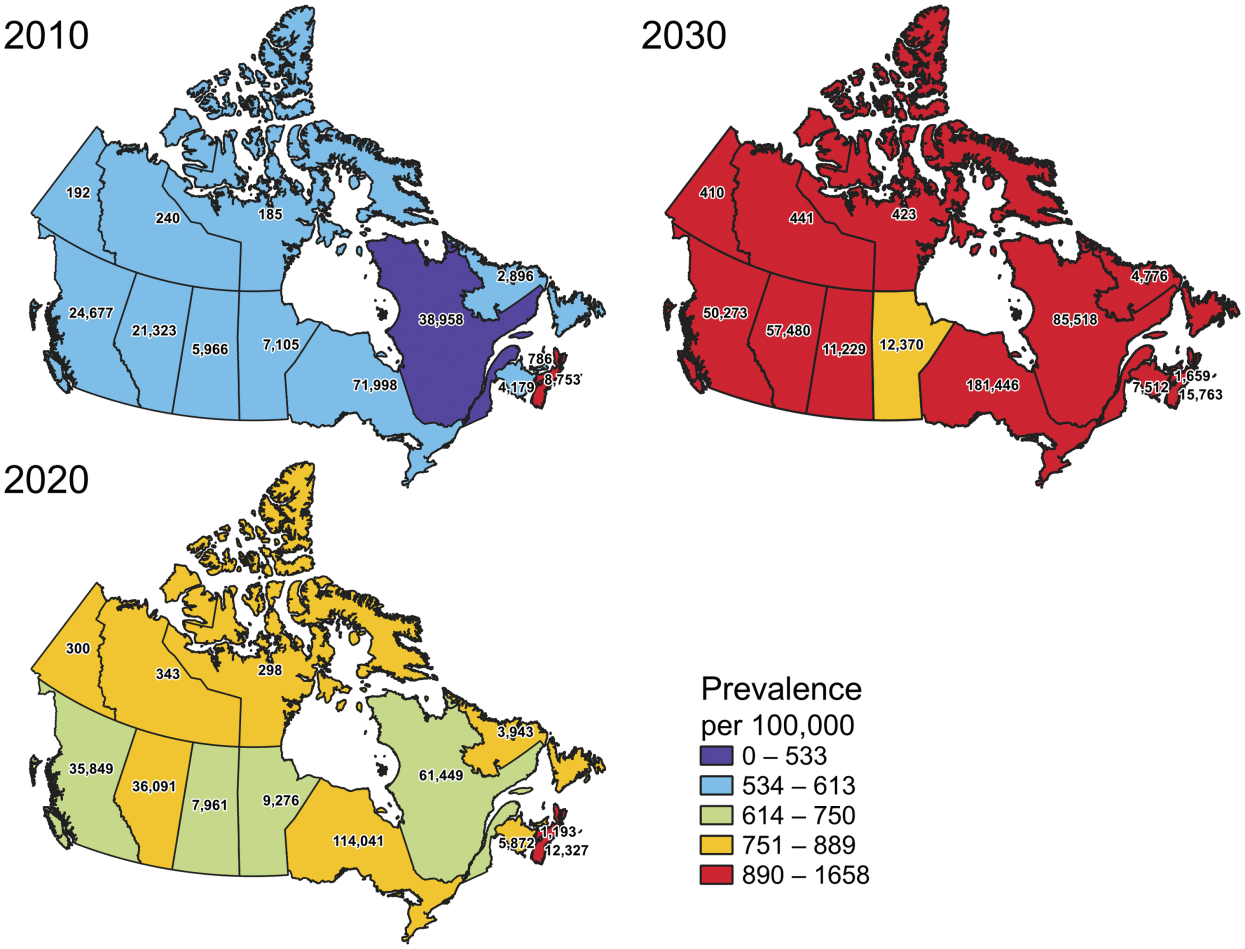
The change in prevalence (colour gradient) of inflammatory bowel disease per 100,000 population and by estimated case numbers (text in provinces) in Canadian provinces and territories in three time periods: 2010, 2020, and 2030. Data on prevalence and cases was estimated from administrative healthcare databases from seven provinces (British Columbia, Alberta, Saskatchewan, Manitoba, Ontario, Quebec, and Nova Scotia) and modeled to estimate provinces/territories without direct data and then forecasted out to 2030 (2).

Roughly 300,000 people are living with inflammatory bowel disease (IBD) in Canada in 2021, and this number is expected to exceed 400,000 by 2030 (1–3). The prevalence of IBD in Canadians is estimated to have risen roughly 50% in the last 10 years (from 0.55% of the Canadian population in 2010 to 0.76% in 2020), and is expected to increase to 1% of the Canadian population by 2030 (Figure 1) (2,4). Seniors (those aged 65+) with Crohn’s disease or ulcerative colitis represent the fastest-growing group of Canadians with IBD and face complications associated with longer disease duration alongside other age-related comorbidities (5,6). On the opposite end of the age spectrum, children with IBD are at risk of unique disease complications, such as impairment of linear growth, and may respond differently to treatments or be at greater risk of related side effects as compared to adults (7).

The World Health Organization (WHO) declared the novel SARS-CoV-2 outbreak a global pandemic on March 11, 2020 (8); this immediately raised concerns among individuals suffering from immune-mediated diseases and their healthcare providers. Given the paucity of knowledge early in the pandemic, the rapid dissemination of information, and the potential susceptibility of immunocompromised people living with IBD, the Scientific and Medical Advisory Council (SMAC) of Crohn’s and Colitis Canada (CCC) instituted a task force to make evidence-based recommendations to people with IBD. In order to deliver expert recommendations and answers to the IBD community, CCC launched a knowledge translation initiative consisting of a webinar series and online resources.

In this article, we detail the dynamic and iterative process of the knowledge translation initiatives developed to inform and protect the IBD community during the first year of the pandemic.

## CROHN’S AND COLITIS CANADA’S COVID-19 AND IBD TASKFORCE

**Figure 2.**
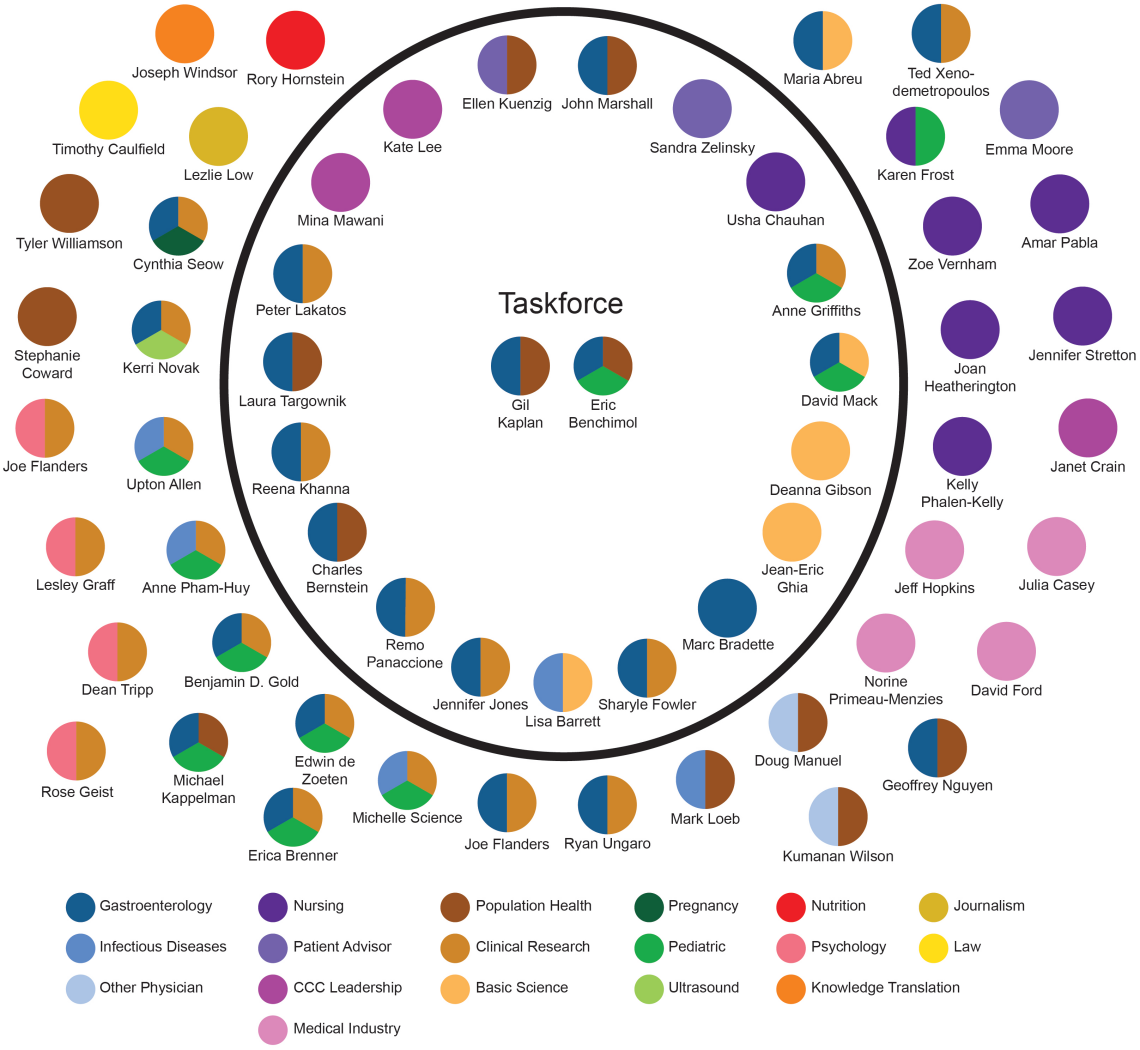
Expertise of the Crohn’s and Colitis Canada COVID-19 and IBD Taskforce (in the circle) and guest panel speakers for webinars (outside of the circle).

On March 12, 2020, the SMAC of CCC met to discuss the COVID-19 pandemic and its potential impact on the IBD community. Together with CCC leadership, the Council agreed that a broader group of experts was necessary to determine recommendations for the IBD community considering the general lack of knowledge on risk factors and scarcity of supporting scientific evidence. On March 17, 2020, the COVID-19 and IBD Taskforce convened via videoconference with representatives from across Canada, including: adult and pediatric gastroenterologists (GIs), IBD nurses, infectious diseases experts, scientists, public health officials, communications and government relations experts, and patient advisors (Figure 2).

The Taskforce met weekly from March 17, 2020 through June 16, 2020 during the first wave of the pandemic in Canada, and reconvened with monthly meetings in September 2020 to address the second wave. The main deliverable of this group was guidance for the IBD community with the caveat that COVID-19 knowledge was evolving rapidly, and recommendations would be reviewed and revised regularly. Topics covered during these online videoconferences largely reflected questions and concerns posed directly by the IBD community and informed the knowledge translation campaign championed by CCC.

Over the course of the pandemic, it became clear that Canada’s COVID-19 epidemiology differed by region. The central prairie region (Manitoba and Saskatchewan) and most of the Atlantic region (New Brunswick, Prince Edward Island, Nova Scotia, and Newfoundland and Labrador) initially had low to medium case counts, but were able to limit the spread of the virus early on and experienced fewer total cases (9). In order to respect the local epidemiology of the pandemic in different jurisdictions within Canada, an emphasis was placed on providing the most up-to-date information available, and encouraging the IBD community to assess their personal risk. However, viewers were urged to contact their own healthcare providers for individual health advice. The general guidance provided considered factors such as age, medications, and comorbid conditions.

## TASKFORCE RECOMMENDATIONS

Recommendations were based on available evidence that included guidelines from gastroenterology societies (e.g., the International Organization for the Study of IBD [IOIBD]), experience from prior viral outbreaks, and current public health guidance modified for the needs of the IBD community (9–11). Recommendations were dynamic as knowledge and global penetrance of SARS-CoV-2 was expanding rapidly; thus, recommendations were frequently updated to reflect new data and were communicated to the IBD community in almost real-time. The Taskforce determined that recommendations should be presented within the context of various risk factors such as: age, comorbidities, status of disease (i.e., new diagnosis, current or recent flare, or remission), and medications (e.g., corticosteroids, biologics). The goal was to offer guidance to the IBD community so that individuals could assess their own IBD profile and minimize their own personal risk.

Recommendations were posted to CCC’s website. An explicit statement that the recommendations should supplement, but not replace, the recommendations made by an individual’s physician or local public health authority was included in all communication. Detailed information made available included FAQ sheets and video clips from a weekly webinar series (Table 1). Over the first six months of the pandemic, recommendations evolved. New evidence emerged regarding risk factors, transmissibility, and medications that may exacerbate negative outcomes from agencies like the Public Health Agency of Canada, the WHO, and Centers for Disease Control in the United States (9,12,13). The breadth of knowledge regarding COVID-19 and IBD-specific risk factors also grew (14,15).

Our understanding of COVID-19 outcomes specific to the IBD community came through multiple data sources (16,17), including the Surveillance Epidemiology of Coronavirus Under Research Exclusion (SECURE-IBD) registry (14,15). SECURE-IBD is a physician self-report database that collects information on global cases of COVID-19 occurring in people with IBD (14,15). The registry data includes: disease type (Crohn’s disease or ulcerative colitis), disease activity (remission, mild, moderate/severe, unknown), age, sex, medications for IBD, country of origin, and outcomes of COVID-19 (recovery, hospitalization, death). An online interactive map displays the data captured in SECURE-IBD (18). The first case was reported to the registry on March 13, 2020, and as of March 19, 2021, 5596 cases have been reported to the registry. Based on the SECURE-IBD data, the most significant risk factors for negative outcomes of COVID-19 were identified as age, active disease (defined by physician global assessment), and prednisone use (14,15). Moreover, people with IBD on biologics did not have increased risk of severe complications of COVID-19 (i.e., need for hospitalization, intensive care, or death).

**Figure 3.**
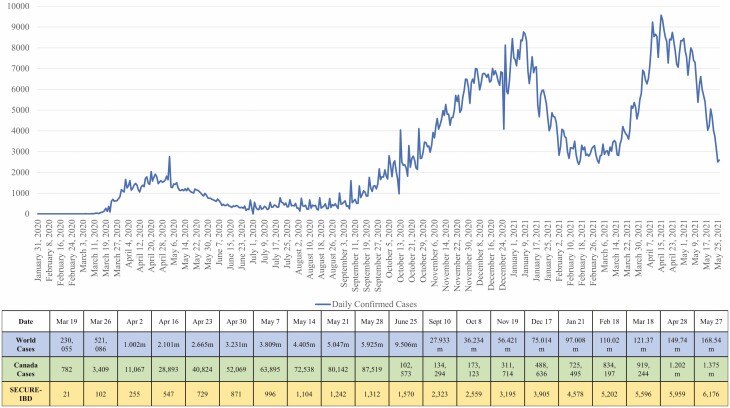
Daily cases of COVID-19 diagnosed in Canada from January 2020 to May 2021 alongside a Table showing the cumulative number of diagnosed COVID-19 in the world, across Canada, and in the Surveillance Epidemiology of Coronavirus Under Research Exclusion (SECURE) IBD registry. Source: Gov. of Canada, Coronavirus disease (COVID-19): Outbreak update—Canada.ca: https://www.canada.ca/en/public-health/services/diseases/2019-novel-coronavirus-infection.html

The evidence from the registry supported a central message of the CCC COVID-19 and IBD Taskforce: People with IBD in clinical remission on medications and without infectious symptoms should not stop their treatments. This message was consistently delivered through the three waves of the pandemic that saw daily cases of COVID-19 diagnosis in Canada peak at over nearly 9000 cases per day in April 2021 (Figure 3).

## WEBINAR SERIES

The core strategy of the knowledge translation initiative was a weekly webinar series moderated by the co-chairs of the CCC COVID-19 and IBD Taskforce (GGK & EIB). These webinars were developed as the primary mechanism to communicate critical information directly to the IBD community in a manner that was accessible to a broad audience. The webinars were promoted through email membership and volunteer lists compiled by CCC, as well as CCC’s social media network (Facebook, Twitter and Instagram). As with the topics for Taskforce discussion, content was developed based on questions received directly from the IBD community in a questionnaire filled out during registration for the webinars, from the live chat during the webinar broadcast, or from the post-webinar surveys deployed to all webinar registrants. The direct connection to people with IBD and their families addressed the critical requirement for effective knowledge translation with information directly relevant to the audience (19).

Questions and concerns were discussed by Taskforce members who collectively determined suitable experts to participate in upcoming webinars as panellists (Figure 2). An illustrative example was the concerns expressed by many individuals regarding infusion clinics very early on in the pandemic: Were they safe, and should those scheduled for infusions keep their appointments? The Taskforce gathered a panel of representatives from infusion clinics across the country to share how they were working together to ensure everyone’s safety through measures that include: Physical distancing (the removal of some infusion chairs to allow for physical distancing during treatment), sanitation, and pre-screening. The resulting webinars were well-received and encouraged the IBD community to express further concerns and topics of importance, including: Mental health; children; pregnancy; risk factors of specific medications; and what to do as businesses, the economy and schools reopened.

The format of every webinar included an introduction by CCC; an update on the epidemiology of COVID-19 by SMAC Chair, Dr. Gilaad Kaplan; an update of the Taskforce recommendations and review of changes to the website by SMAC Chair-Elect, Dr. Eric Benchimol; followed by the topic segment, usually a guest presentation on the topic and a panel discussion with experts. After each webinar, a pertinent discussion was selected to produce two-to-five-minute segments and posted online.

The epidemiologic update included weekly presentations using data from Johns Hopkins University to illustrate the global epidemiology of COVID-19 (20), data from Public Health Agency of Canada that illustrated the details (including health outcomes) of confirmed Canadian cases of COVID-19 (9), and an update from the SECURE-IBD registry that illustrated IBD-specific cases of COVID-19 (14,15,18). The epidemiology update was usually followed with one or two case studies prepared for a lay audience to illustrate a key piece of evidence, such as projected possible waves of COVID-19 in comparison to the 1918 influenza pandemic, or case studies that highlight risk of virus transmission. After the epidemiology and Taskforce recommendation updates, webinars focused on a particular topic in an episodic nature where a panel of experts was invited to give presentations to the audience or have a virtual round-table discussion. The topics covered in each of the webinars, as well as the confirmed COVID-19 cases globally, in Canada, and in SECURE-IBD at the time of each webinar are presented in Table 2.

**Table 2.** Date-specific webinar topics with liver registrants and archived video views

Date	Topic	Registrants*	Live Views	Archived Video Views*
19/3/2020	COVID-19 and IBD: What You Need to Know	2679	1558	8775
26/3/2020	Infusion Clinics, Pregnancy and Newborns	1269	667	2697
2/4/2020	COVID-19 Risk Factors, Live Q&A	2169	987	2483
9/4/2020	COVID-19 Updates, Mental Health, Diet and Nutrition	992	480	1364
16/4/2020	Modeling COVID-19 to Prepare for the Future	762	342	795
23/4/2020	Monitoring COVID-19 in IBD Persons (SECURE IBD registry)	843	409	1210
30/4/2020	Telemedicine in the Pandemic	710	346	515
7/5/2020	Families and Children with IBD	751	369	764
14/5/2020	COVID-19 Risk Factors, Reopening of School and the Economy	557	293	678
21/5/2020	COVID-19 Updates, Q&A with IBD Nurses	551	275	540
28/5/2020	Mental Health and Wellness	439	204	429
4/6/2020	Washroom Access and Reopening of Businesses	382	174	433
11/6/2020	IBD Clinic of the Future	420	174	367
18/6/2020	IBD Medications and COVID-19 Risk	500	220	2347
25/6/2020	Separating COVID-19 Myths from the Facts	524	195	1690
10/9/2020	Returning to school for families with IBD	598	251	802
8/10/2020	Vaccinations and IBD	1542	541	1867
19/11/2020	SECURE-IBD Registry	710	253	641
07/12/2020	Les MII et la COVID-19 : Ce que l’on a appris et les questions qui restent	573	315	606
17/12/2020	Vaccines and “Home” for the Holidays	2672	1058	1611
21/01/2021	New Recommendations: COVID-19 Vaccine for IBD	3112	1360	3054
18/02/2021	COVID-19 Updates and Hitting the Pandemic Wall	968	471	926
18/03/2021	A Year in Review and Vaccine Updates	1209	604	1220

*Estimates as of April 1, 2021. Total registrants: 24,778; total live audience: 11,511; total archived video views: 35,814

A detailed Frequently Asked Questions document was developed from the webinar presentations that was curated into a web-based information source on the CCC website. The answers to the questions contained links to pertinent clips from webinars in order to provide more detailed information and an alternate form of information delivery. The webinars were archived on CCC’s YouTube channel, and on CCC’s webpage. View counts of the archived videos were typically four to five times those of the live webinars (Table 2). For specific topics related to recommendations made on the guidance webpages, webinar videos were spliced into segments of 5 min or less and embedded next to the recommendations on the webpage; this allowed readers of the webpage to watch the related webinar segment with experts discussing the reasoning and scientific evidence behind the recommendations.

## IMPACT

The webinars and digital CCC resources were promoted through social media and email notifications to the IBD community across Canada. The 15 weekly, seven monthly webinars, as well as the one French-language webinar saw a total of 24,778 registrations with one-third (33%) registering for more than one COVID19 webinar. Links to the recorded webinars were provided on the CCC website 24 h after the live event and added to the organization’s YouTube page. Archived webinars were also captioned in French and recent webinars offer live French translations. A further 35,814 views of the full recordings of the webinars have been tallied to date (April 1, 2021). The ability to select pieces of each video to augment COVID-19 information on the CCC website has proven to be exceptionally impactful with a further 78,862 views of individual clips (Table 2). As of April 1, 2021, there have been 54,136 views of the webinars (live or recorded full webinars) and 78,862 views of individual webinar segments for a total of 126,187 views. Traffic on CCC’s website increased dramatically since COVID-19, with 484,755 unique views to the COVID-19 web pages. The visitors spent between 0.10 and 28.29 min on the pages with a mean duration of 40.29 s.

**Table 1.** Recommendations developed by Crohn’s and Colitis Canada COVID and IBD Taskforce and associated links to website page (https://crohnsandcolitis.ca/About-Crohn-s-Colitis/COVID-19-and-IBD)

Title	Description	Web-link
General information on COVID-19	Explanation of COVID-19, epidemiology, symptoms and outcomes.	https://crohnsandcolitis.ca/About-Crohn-s-Colitis/COVID-19-and-IBD/What-is-COVID-19
Visiting clinics and testing	Discusses visiting physicians, diagnostic investigation (e.g., bloodwork) and infusion clinics.	https://crohnsandcolitis.ca/About-Crohn-s-Colitis/COVID-19-and-IBD/Clinic-Visits-and-Testing
Diet and nutrition	Reviews diets and nutritional needs for persons with IBD, as well as grocery shopping and restaurants during the pandemic.	https://crohnsandcolitis.ca/About-Crohn-s-Colitis/COVID-19-and-IBD/Diet-and-Nutrition
Your wellbeing	Overviews mental health and wellness, including mechanism for coping with stress and anxiety.	https://crohnsandcolitis.ca/About-Crohn-s-Colitis/COVID-19-and-IBD/Mental-Health-and-Wellness
Information for health professionals	Section for healthcare professionals including an overview of the SECURE-IBD Registry and resources for their persons with IBD.	https://crohnsandcolitis.ca/About-Crohn-s-Colitis/COVID-19-and-IBD/For-Professionals
Guidance/Recommendations		
Travel and physical distancing	Discusses restrictions on travel and appropriate physical distancing during the pandemic.	https://crohnsandcolitis.ca/About-Crohn-s-Colitis/COVID-19-and-IBD/Guidance/Travel-and-Physical-Distancing
Risk profile	Provides self-assessment risk profile with corresponding recommendations.	https://crohnsandcolitis.ca/About-Crohn-s-Colitis/COVID-19-and-IBD/Guidance/Are-you-at-Risk
Medications for IBD	Explains the risk associated with medications for IBD.	https://crohnsandcolitis.ca/About-Crohn-s-Colitis/COVID-19-and-IBD/Guidance/Medications
Children and teens	Reviews the risk associated with COVID-19 for children and adolescents with IBD. Discussion of risks associated with returning to in-person school.	https://crohnsandcolitis.ca/About-Crohn-s-Colitis/COVID-19-and-IBD/Guidance/Children-with-IBD
Reopening of the economy	Provides guidance for adults and seniors with IBD as the lockdown ended and the economy re-opened.	https://crohnsandcolitis.ca/About-Crohn-s-Colitis/COVID-19-and-IBD/Guidance/Reopening-of-the-Economy
Essential work and services	Discusses special considerations for persons with IBD who are involved with essential work (e.g., healthcare providers).	https://crohnsandcolitis.ca/About-Crohn-s-Colitis/COVID-19-and-IBD/Guidance/Essential-Work
Tested positive for COVID-19	Information for individuals with IBD who test positive for COVID-19.	https://crohnsandcolitis.ca/About-Crohn-s-Colitis/COVID-19-and-IBD/Guidance/If-you-have-COVID-19
Pregnancy and newborns	Overviews recommendations for persons with IBD who are pregnant or have a newborn.	https://crohnsandcolitis.ca/About-Crohn-s-Colitis/COVID-19-and-IBD/Guidance/Pregnancy-and-Newborns
Family members of patients with IBD	Explains actions that family members can take to support their household member with IBD.	https://crohnsandcolitis.ca/About-Crohn-s-Colitis/ COVID-19-and-IBD/Guidance/Family-Members- of-People-with-IBD
COVID-19 vaccines	Describes CCC recommendation regarding vaccination for persons with IBD for Health Canada approved COVID-19 vaccines.	https://crohnsandcolitis.ca/About-Crohn-s-Colitis/COVID-19-and-IBD/Vaccines
Frequently asked questions	Summary of frequently asked questions based on surveys and feedback from the IBD community during the pandemic.	https://crohnsandcolitis.ca/About-Crohn-s-Colitis/COVID-19-and-IBD/Get-Answers
Webinars	Information on the next webinar and option for registration. Webpage houses all previously recorded webinars on COVID-19 and IBD.	https://crohnsandcolitis.ca/About-Crohn-s-Colitis/COVID-19-and-IBD/COVID-19-Webinars

## CONCLUSION

CCC was able to quickly assemble the COVID-19 and IBD Taskforce at the outset of the global pandemic. The Taskforce members have met and continue to meet regularly in an effort to ensure that the IBD community has the best available information to support them as they navigate a new reality with COVID-19. Direct communication from the Taskforce and the expert community in Canada to people with IBD and caregivers through a webinar series was an effective and efficient knowledge translation vehicle. The spring webinars ably guided the vulnerable IBD community from a population-wide lockdown in March 2020 through to an understanding of risk and appropriate measures to ensure physical and mental health during the re-opening of the country over the summer and through the second wave of the pandemic. On March 18, 2021, the one-year anniversary of the webinar series, CCC completed the twenty-third webinar that addressed questions by the IBD community on vaccination. As future waves of the pandemic unfold in Canada, the Taskforce is prepared to guide the IBD community.
